# Platypnoea-orthodeoxia syndrome following transcatheter aortic valve implantation complicated by aortic annular rupture: a case report

**DOI:** 10.1093/ehjcr/ytaf418

**Published:** 2025-08-28

**Authors:** Stuart K Gibson, Arpudh Anandaraj, Christine F McDonald, Anoop N Koshy, Elizabeth Jones

**Affiliations:** Department of Cardiology, Austin Health, 145 Studley Road, Heidelberg, Victoria 3084, Australia; Department of Cardiology, Austin Health, 145 Studley Road, Heidelberg, Victoria 3084, Australia; Department of Respiratory and Sleep Medicine, Austin Health, 145 Studley Road, Heidelberg, Victoria 3084, Australia; Institute for Breathing and Sleep, Austin Health, 145 Studley Road, Heidelberg, Victoria 3084, Australia; Department of Cardiology, Austin Health, 145 Studley Road, Heidelberg, Victoria 3084, Australia; Department of Cardiology, Austin Health, 145 Studley Road, Heidelberg, Victoria 3084, Australia

**Keywords:** Aortic stenosis, Platypnoea-orthodeoxia, Transcatheter aortic valve implantation, Structural intervention, Case report

## Abstract

**Background:**

Platypnoea-orthodeoxia syndrome (POS) is a rare disorder characterized by dyspnoea and hypoxaemia occurring while upright and improving while recumbent, most often caused by inter-atrial shunting. Diagnosis is challenging, and the syndrome carries considerable morbidity if left untreated. In patients with inter-atrial communications such as a patent foramen ovale (PFO), haemodynamic and structural changes occurring after transcatheter aortic valve implantation (TAVI) can lead to platypnoea-orthodeoxia.

**Case summary:**

A 77-year-old patient with severe aortic stenosis was referred for TAVI at our institution. On transthoracic echocardiography, dilatation of the ascending aorta (40 mm) and a mobile atrial septal aneurysm (ASA) were noted. Transcatheter aortic valve implantation was complicated by annular rupture and aortic intramural haematoma (IMH), managed conservatively. Three weeks after discharge, the patient re-presented with cholecystitis and underwent cholecystectomy. Post-operatively, marked hypoxaemia was noted during orthostasis. Transthoracic echocardiography showed the ASA bulging into the left atrium, with a strongly positive saline contrast study while upright. A PFO was identified on transoesophageal echocardiography and successfully percutaneously closed, with hypoxaemia resolving.

**Discussion:**

Platypnoea-orthodeoxia syndrome is a rare complication of TAVI, not previously reported after annular rupture. Aortic dilatation is often implicated in platypnoea-orthodeoxia, likely by compressing the interatrial septum and potentiating right-to-left shunting. In our patient, with a mildly dilated aorta prior to intervention, annular rupture and IMH may have further distorted atrial anatomy. Furthermore, rapid improvements in left ventricular compliance and left-sided filling pressures after TAVI may have facilitated the new onset of right-to-left shunting. Platypnoea-orthodeoxia syndrome should be considered in patients with unexplained hypoxaemia after TAVI.

Learning points

- Platypnoea-orthodeoxia syndrome should be considered as a possible cause of unexplained hypoxaemia after *trans*-catheter aortic valve implantation, as the condition is diagnostically challenging and associated with significant morbidity if untreated.
- The mechanism of platypnoea-orthodeoxia syndrome after transcatheter aortic valve implantation may be explained by marked improvements in left-sided filling pressures, coupled with distortion of atrial anatomy in patients with coexistent aortic dilatation.

## Introduction

Platypnoea-orthodeoxia syndrome (POS) is a rare disorder characterized by dyspnoea and hypoxaemia occurring while upright and improving while recumbent.^1^ Diagnosis is challenging, and the syndrome carries considerable morbidity if left untreated. For the proposed mechanism of positional right-to-left shunting to occur, specific physiological parameters must be present. Firstly, this requires an anatomical substrate for intrapulmonary or intracardiac shunting. In cardiac POS, this is either a patent foramen ovale (PFO), an atrial septal defect (ASD), or a fenestrated atrial septal aneurysm (ASA).^2^ This must be coupled with a functional substrate, either congenital or acquired, that allows preferential shunting of venous blood through the defect, including (but not limited to) aortic root dilatation, tricuspid regurgitation, or a prominent eustachian valve. We report the case of POS in a 77-year-old patient who presented with persistent orthodeoxia after transcatheter aortic valve implantation (TAVI), which was complicated by aortic annular rupture and intramural haematoma (IMH).

## Summary figure

**Figure:**
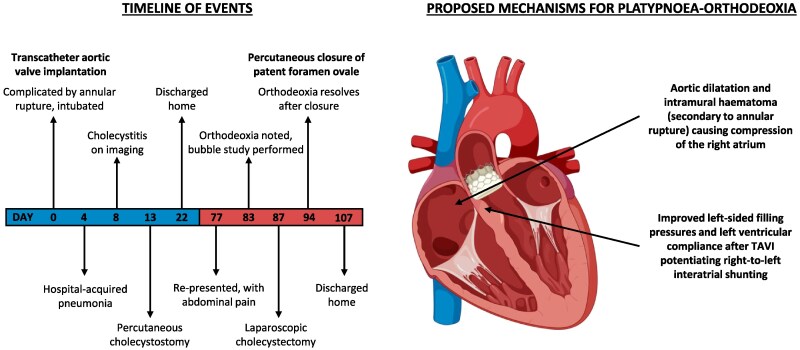


## Case presentation

A 77-year-old patient with severe aortic stenosis (AS) (mean gradient 46 mmHg, aortic valve area 1.0 cm^2^) was referred for TAVI at our institution. His medical history included gout, prostate cancer, and hyperlipidaemia. There was no history of platypnoea nor of cyanotic episodes nor of documented hypoxaemia. Mild fusiform dilatation of the ascending aorta (40 mm) with a tortuous thoracic aorta was noted on computed tomography (CT), and a mobile ASA was visualized on transthoracic echocardiography (TTE). No interatrial shunt was detected on colour Doppler interrogation. However, saline contrast imaging was not performed.

The TAVI procedure (26 mm SAPIEN 3 Ultra Valve) was complicated by a contained annular rupture and aortic IMH, requiring urgent intubation and vasopressor support. The rupture was managed conservatively. On Day 4, the patient developed hypoxia, tachypnoea, and fevers and was treated for hospital-acquired pneumonia with piperacillin-tazobactam. With fevers persisting, CT aortogram/chest/abdomen/pelvis was performed, demonstrating aortic IMH, bilateral pleural effusions, and features of acute cholecystitis, which was managed with percutaneous cholecystostomy. Transthoracic echocardiography confirmed a well-seated aortic valve prosthesis with mild paravalvular regurgitation and a small pericardial effusion without features of tamponade. The hypoxaemia improved over several days and he was discharged home.

**Figure 1:**
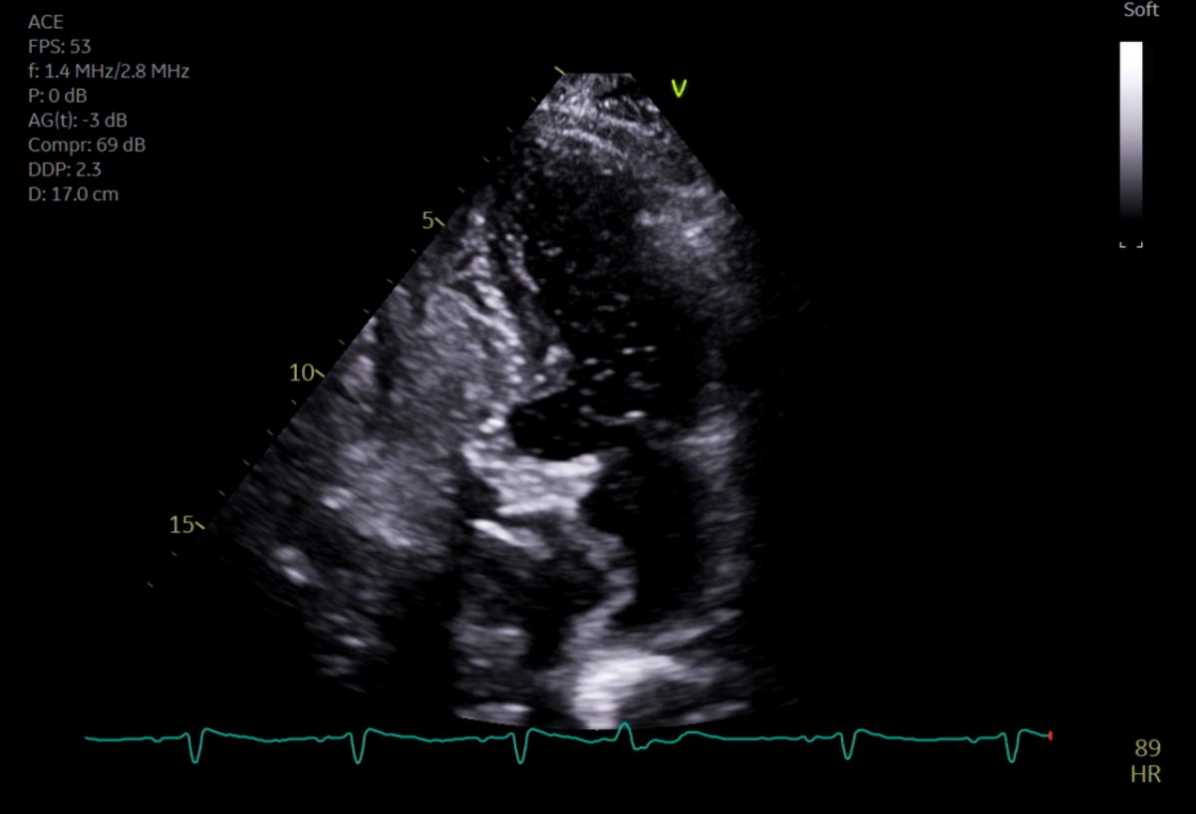
Transthoracic apical four-chamber image obtained in the upright position. The atrial septum is aneurysmal and bows towards the left atrium, suggesting an interatrial pressure gradient. Intravenous agitated saline has been administered and opacifies the right atrium and ventricle. Saline microbubbles are noted in the left ventricle, consistent with an intracardiac right to left shunt.

The patient represented 8 weeks later with fevers and abdominal pain, and cholecystectomy was planned. Preoperatively, recurrent profound episodes of hypoxaemia were noted, requiring prolonged non-invasive ventilation, with PaO_2_ of 63 mmHg on FiO_2_ of 0.4, demonstrating an elevated A-a gradient of 163 mmHg. On examination, blood pressure was 139/63 mmHg, pulse was 99 beats per minute, and respiratory rate was 20 breaths per minute. The peripheries were warm, there were no cardiac murmurs, and the chest was clear to auscultation. Intravenous furosemide did not improve the hypoxaemia, and chest X-ray demonstrated no interstitial oedema. Interestingly, although the patient was able to ambulate without dyspnoea, marked hypoxaemia (nadir saturation 81%) was noticed during orthostasis, raising the possibility of POS. Computed tomography pulmonary angiography found no evidence of pulmonary emboli or pulmonary arteriovenous malformations to cause intra-pulmonary shunting. Repeat TTE was performed to investigate potential cardiac POS. While this demonstrated resolution of the pericardial effusion, colour Doppler interrogation showed a right-to-left interatrial shunt, and a saline contrast study was positive in the upright position (*Figure 1*). There was no evidence of elevated right heart pressures other than bowing of the ASA into the left atrium (LA), and the shunt was thought unlikely to be the cause of profound hypoxaemia. The patient underwent laparoscopic cholecystectomy. However, post-operatively, the hypoxaemia worsened, with clear orthodeoxia. As no cause other than interatrial shunting was identified, percutaneous closure of the PFO was scheduled.

**Figure 2:**
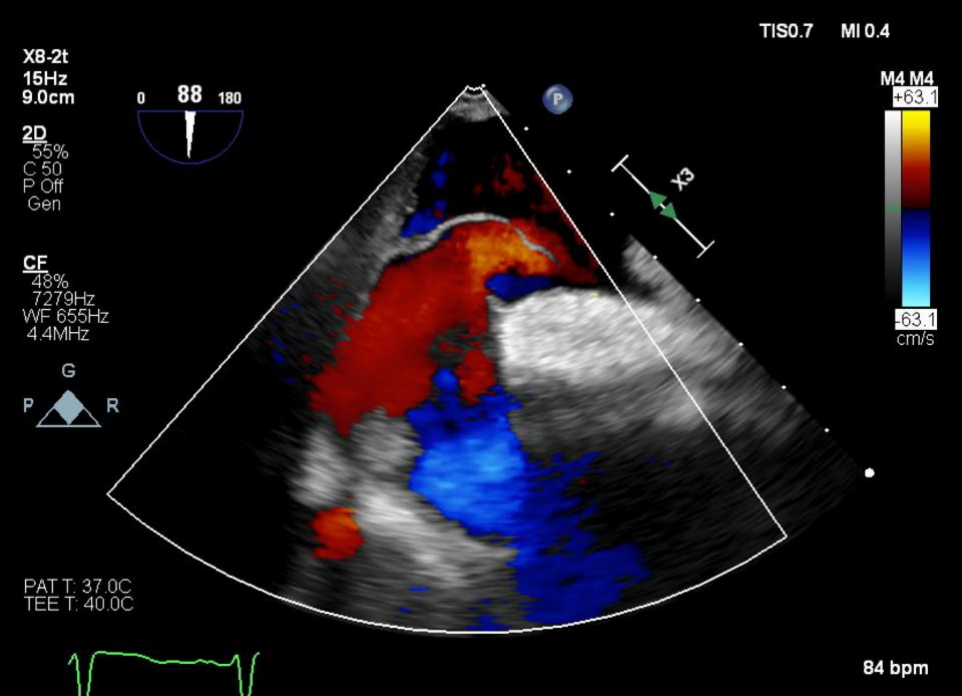
Mid-oesophageal–modified bicaval view. The atrial septum is aneurysmal and bows towards the left atrium. Streaming of inferior vena cava flow into the right atrium is demonstrated. Flow passes into the left atrium through a widely patent foramen ovale. Normal flow from the right atrium to right ventricle is denoted in blue.

**Figure 3:**
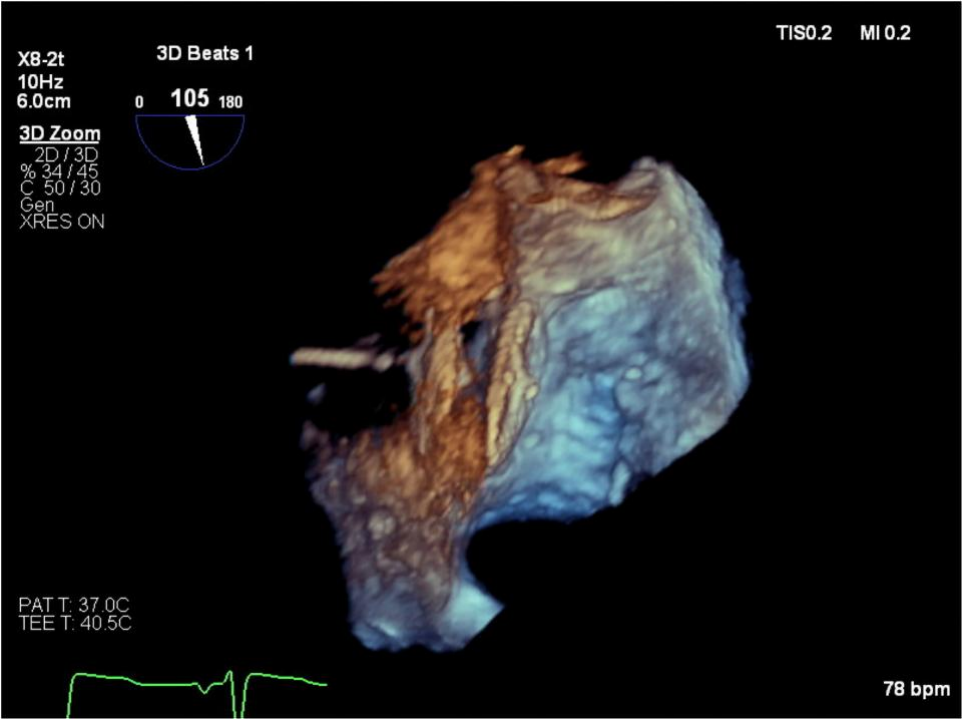
3D transoesophageal image of the patent foramen ovale occluder device *in situ* before release. The atrial septum is captured between the 30 mm right atrial disc and the 25 mm left atrial disc of the device, thus stabilizing the atrial septal aneurysm, closing the foramen ovale and eliminating the right-to-left shunt.

**Figure 4:**
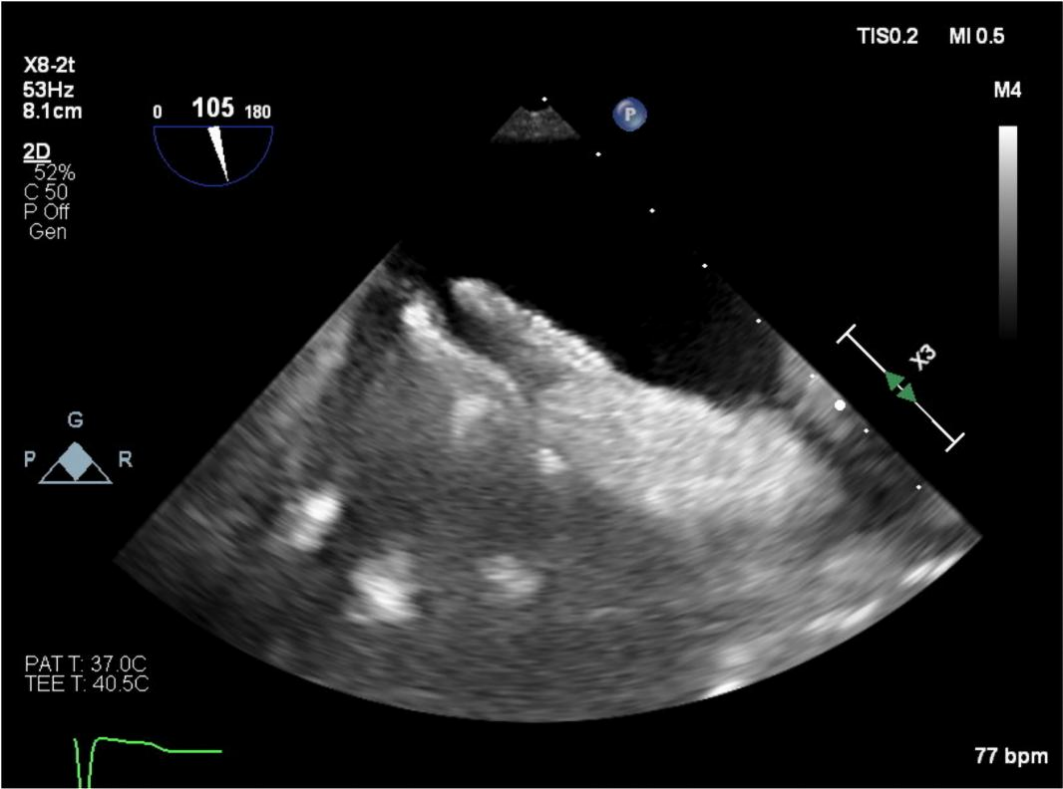
Mid-oesophageal bicaval view demonstrating satisfactory Amplatzer PFO Occluder device position following deployment. The discs of the device are parallel, and there is no encroachment on the aortic root anteriorly or the atrial wall or pulmonary veins posteriorly.

Intra-procedural TOE demonstrated a thickened aortic wall consistent with prior haematoma and an aneurysmal interatrial septum with large PFO, opening to 6 mm (*Figure 2*). Prior to closure, the mean pulmonary artery pressure was invasively measured at 18 mmHg, excluding pulmonary hypertension as a cause of shunting. The PFO was successfully closed with a 30/25 mm Amplatzer PFO Occluder (*Figures 3* and *4*). Arterial oxygen saturations improved immediately from 94% to 99%. Several months later, the patient has no dyspnoea, with repeat TTE confirming mild-to-moderate paravalvular aortic regurgitation and no residual shunt.

## Discussion

Platypnoea-orthodeoxia syndrome is a rare complication of TAVI and has not previously been reported after aortic annular rupture. Diagnosing cardiac POS is challenging and typically requires demonstration of orthostatic arterial hypoxaemia, a positive bubble study for right-to-left shunting while upright (with the appearance of bubbles in the LA within three cardiac cycles indicating an intracardiac aetiology), and echocardiographic visualization of an anatomical substrate (e.g. PFO or ASD) for shunting. Percutaneous closure of PFO or ASD is often preferred, with decreased morbidity compared to surgical intervention.^2^ However, while these substrates for interatrial shunting are not uncommon, these defects alone do not cause cardiac POS; a second, functional abnormality must distort cardiac anatomy during orthostasis.^2,3^ With the precise pathophysiology for cardiac POS remaining unclear, this case illustrates how haemodynamic changes caused by TAVI coupled with aortic root dilatation and distortion by annular rupture may have provoked positional right-to-left shunting in our patient.

Platypnoea-orthodeoxia syndrome is often associated with aortic pathology, with Eicher *et al*.^4^ identifying a dilated aortic root as the likely aetiology in 42% of POS cases. Our patient had prior aortic dilatation, which frequently coexists with severe AS and is present in one-fourth of patients undergoing TAVI, particularly those with a bicuspid aortic valve.^5,6^ However, our patient’s degree of aortic dilatation was significantly below the threshold (≥50 mm) for surgical intervention, as per European Society of Cardiology (ESC) guidelines.^7^ To determine the relationship between PFO and aortic pathology, Bertaux *et al*.^8^ used echocardiography to demonstrate that aortic dilatation compressed the interatrial septum (IAS) which, in the presence of PFO, potentiated right-to-left shunting, while Laybourn *et al*.^9^ proposed that ascending aortic enlargement distorted caval inflow towards the interatrial septum. The significance of acute annular rupture and aortic intramural haematoma formation in our patient is unclear. However, it is plausible that this may have exacerbated compression of the right atrium (RA) caused by prior aortic dilatation, thus contributing to distortion of the IAS and subsequent orthodeoxia.

The haemodynamic changes observed following TAVI may also have caused POS in our patient. Severe AS results in elevated left-sided filling pressures and left atrial dilatation.^10^ Following TAVI, left ventricular compliance and diastolic pressures improve, favouring right-to-left shunting. Reverse remodelling and improvements in ejection fraction have been observed 30days after TAVI in cardiac MRI studies;^11^ orthostatic right-to-left shunting may have become haemodynamically possible in our patient after a period of left atrial and ventricular reverse remodelling. This mechanism could also explain why orthodeoxia was not apparent until several weeks post-TAVI.

Platypnoea-orthodeoxia syndrome is a rare but important cause of hypoxaemia and is associated with high morbidity if untreated. Patients with PFO or ASD and aortic pathology (either pre-existing or, as in this case, iatrogenic) are predisposed to interatrial shunting. When coupled with profound haemodynamic and physiological changes following TAVI, platypnoea-orthodeoxia may occur.

## Lead author biography

Dr Stuart K. Gibson graduated from Newcastle University School of Medicine (UK) in 2020. He is currently a physician trainee at Austin Health, Melbourne, with an interest in arrhythmia and has been accepted for a Cardiology Advanced Trainee position in 2026.
